# Effects of occupational therapy on psychiatric symptoms and social functioning in patients with schizophrenia: a systematic review and meta-analysis

**DOI:** 10.3389/fpsyt.2026.1862106

**Published:** 2026-08-12

**Authors:** Liping Sun, He Teng, Liru Zhang, Xuebin Gao, Wenhang Li, WenLong Jiang, Linlin Song, Qian Xu

**Affiliations:** 1 Jiamusi University, Jiamusi, Heilongjiang, China; 2 Daqing Third Hospital, Daqing, Heilongjiang, China

**Keywords:** meta-analysis, occupational therapy, psychiatric symptoms, schizophrenia, systematic review

## Abstract

**Objective:**

The present study aimed to systematically evaluate the impact of occupational therapy on the psychiatric symptoms and social functions of patients with schizophrenia.

**Methods:**

A comprehensive literature search was conducted in CNKI, Wanfang Data, VIP Database, PubMed, Web of Science, the Cochrane Library, and Embase from 2015 to February 2026. Randomized controlled trials (RCTs) of occupational therapy assessing its impact on mental symptoms and social functioning in patients with schizophrenia were included. The following data were extracted: author, year, age, country, measures for the control group and the intervention group, diagnostic criteria, sample size, and outcomes. Meta-analysis was performed using RevMan 5.4 and R 4.4.3 software. The primary outcome was psychiatric symptom severity assessed by the total score of the Positive and Negative Syndrome Scale (PANSS), whereas secondary outcomes included social functioning measured by the Social Disability Screening Schedule (SDSS), behavioral and psychiatric symptoms evaluated by the Nurses’ Observation Scale for Inpatient Evaluation (NOSIE), and activities of daily living assessed using the Activities of Daily Living (ADL) scale. Fixed or random-effects models were applied according to heterogeneity, and subgroup analyses were conducted.

**Result:**

A total of 10 studies, conducted in China and Japan, were eventually included in this meta-analysis, involving 958 participants (476 in the intervention group and 482 in the control group). The meta-analysis revealed that the occupational therapy group had a significantly lower total PANSS score than the conventional treatment group, suggesting that occupational therapy may contribute to the alleviation of psychiatric symptoms (MD: − 5.76; 95% CI, − 7.72 to − 3.79; *p* < 0.00001). In terms of social functioning, occupational therapy also demonstrated positive effects, with significantly lower SDSS scores (MD: − 1.06; 95% CI, − 1.94 to − 0.18; *p* = 0.02) and ADL scores (MD: − 3.50; 95% CI, − 5.20 to − 1.79; *p* = 0.0001) in the intervention group compared with the control group, as well as a significantly higher NOSIE score (MD: 32.33; 95% CI, 16.37 to 48.30; *p* < 0.0001), indicating that occupational therapy may improve patients’ daily living abilities.

**Conclusion:**

Occupational therapy-based interventions may contribute to improvements in psychiatric symptoms and appear to exert positive effects on social functioning and activities of daily living in patients with schizophrenia.

**Systematic review registration:**

https://www.crd.york.ac.uk/prospero/, CRD420261341828.

## 1 Introduction

Schizophrenia is a chronic mental disorder with complex etiology and a high disability rate. According to the World Health Organization, the global lifetime prevalence of schizophrenia is approximately 0.5%–1.0%, affecting more than 24 million individuals worldwide (1, 2). The disorder typically has its onset in late adolescence or early adulthood and is characterized by abnormalities in perception, thought, emotion, and behavior, as well as incoordination of mental activities, with a protracted disease course. Approximately half of all patients eventually develop psychiatric disability, imposing a heavy burden on families and society (3). This condition is often accompanied by social dysfunction, decline in activities of daily living, and long-term cognitive impairment. Antipsychotic medications, as the cornerstone of pharmacotherapy, effectively control acute-phase positive symptoms and reduce the risk of relapse (4), remaining a fundamental approach in the comprehensive management of schizophrenia. However, clinical evidence indicates that pharmacotherapy alone yields limited efficacy in improving negative symptoms, cognitive deficits, social dysfunction, and activities of daily living (5). Even after symptom control, many patients encounter difficulties in returning to family and community life, with varying degrees of social impairment that severely affect quality of life and increase the risk of relapse and rehospitalization (6). Furthermore, negative symptoms and adverse effects of long-term medication compromise treatment adherence (7). Therefore, pharmacotherapy alone is insufficient to effectively manage psychiatric symptoms and restore social functioning, and nonpharmacological interventions should be integrated into a comprehensive rehabilitation plan.

Occupational therapy is a professional intervention that utilizes meaningful daily activities as a means of helping patients rebuild life functioning and social roles. The World Federation of Occupational Therapists (WFOT) explicitly states that occupational therapy improves individual physical and mental health by guiding individuals to engage in purposeful occupational activities (8). Theoretically, occupational therapy is grounded in three classic models—the Model of Human Occupation (MOHO), the Person–Environment–Occupation (PEO) model, and the Canadian Model of Occupational Performance and Engagement (CMOP-E). This framework aligns with the World Health Organization’s International Classification of Functioning, Disability and Health (ICF), addressing patients’ functional deficits across multiple dimensions, including physical function, daily activities, social participation, and environmental adaptation (9). Compared with broad psychosocial rehabilitation approaches, occupational therapy has a distinct nature: conventional psychosocial rehabilitation typically provides general psychological support, health education, and ordinary group activities to help patients adapt to their environment, whereas the core of occupational therapy lies in engaging patients in meaningful, structured activities, such as activities of daily living training, social skills training, cognitive training, and leisure activities, to restore their daily living abilities and social adaptive capacity. The American Occupational Therapy Association (AOTA) (10, 11), in its fourth edition of the Occupational Therapy Practice Framework, further delineates eight core domains of intervention, encompassing activities of daily living, instrumental activities of daily living, health management, rest and sleep, education, work, leisure, and social participation.

Previous studies have confirmed the beneficial effects of occupational therapy on patients with schizophrenia. Activities of daily living training, such as cooking, shopping, and time management, helps patients regain independent living skills (12). Group activities, including handicrafts, painting, gardening, and music, provide social contexts that facilitate communication, cooperation, and emotional expression, thereby improving social skills (13). Cognitive-oriented activities can train attention, memory, executive function, and problem-solving abilities, consequently ameliorating cognitive deficits (14, 15). Vocational behavior training, through simulated work scenarios and vocational skill instruction, enhances job readiness and facilitates return to work (16, 17), while also bolstering self-efficacy and a sense of meaning in life, thus improving overall mental health status (18, 19). In recent years, several randomized controlled trials have been conducted internationally (20, 21), such as individualized occupational therapy for severe mental disorders at a tertiary medical center in South India (22) and a controlled trial examining the effects of rehospitalization on functional remission and subjective recovery in patients with schizophrenia (23).

Despite preliminary evidence supporting the rehabilitative value of occupational therapy, substantial evidence gaps remain in this field. Considerable variation exists across studies in intervention details and treatment duration, with no unified standardized protocol yet established. Most available studies are small-sample independent trials, lacking large-scale pooled analyses. A systematic review published in 2025 summarized only the cognitive benefits of occupational therapy (15), without examining the relationship between intervention format, treatment duration, and therapeutic efficacy. More importantly, existing evidence does not provide a comprehensive evaluation of psychiatric symptoms, social functioning, and activities of daily living as outcome measures. Thus, questions regarding the differential efficacy of various intervention modalities and treatment durations remain unresolved. To address these evidence gaps, the present study aimed to systematically evaluate the effects of occupational therapy on psychiatric symptoms and social functioning in patients with schizophrenia, thereby providing a scientific basis for comprehensive rehabilitation strategies and offering references for the application and dissemination of occupational therapy in psychiatric settings.

## 2 Methods

### 2.1 Registration and protocol

The meta-analysis was reported by the Preferred Reporting Items for Systematic Reviews and Meta-Analyses (PRISMA) statement (24). The study protocol was registered with PROSPERO (CRD420261341828).

### 2.2 Search strategy

The PubMed, Cochrane Library, Embase, Web of Science, Wanfang Data Knowledge Service Platform, CQVIP, and China National Knowledge Infrastructure (CNKI) databases were searched for randomized controlled trials (RCTs) published between 2015 and February 2026. No language restrictions were applied. The following words and MeSH terms were used: “schizophrenia” and “occupational therapy”. The complete PubMed search strategy was as follows: (schizophrenia[MeSH Terms] OR schizophrenia[Title/Abstract] OR schizophreniform disorder[Title/Abstract]) AND (Occupational Therapy[MeSH Terms] OR occupational therapy[Title/Abstract] OR work therapy[Title/Abstract]) AND 2015:2026[dp]. The detailed search strategy is presented in **Supplementary Table 2**.

### 2.3 Inclusion and exclusion criteria

The inclusion criteria were as follows: (1) Population: patients aged ≥ 18 years who were diagnosed with schizophrenia according to any of the following diagnostic criteria: the International Classification of Diseases, 10th Revision (ICD-10), the Chinese Classification and Diagnostic Criteria of Mental Disorders, Third Edition (CCMD-3), or the Diagnostic and Statistical Manual of Mental Disorders, Fourth or Fifth Edition (DSM-IV or DSM-5),. (2) Intervention: occupational therapy-related interventions delivered to the experimental group, including, but not limited to, individual occupational therapy, group occupational therapy, and combined individual and group occupational therapy. The intervention content was required to fall within the scope of the eight core domains of occupational therapy. Given that most original studies did not fully report the qualifications of intervention providers, therapist credentials were not imposed as a mandatory inclusion criterion. However, for studies that explicitly reported provider qualifications, this information was extracted and summarized in **Supplementary Table 1**. (3) Comparison: the control groups received routine psychiatric care, including conventional treatment (e.g., standard pharmacotherapy and nursing care) or simplified group occupational therapy. (4) Outcome indicators: the primary outcome was psychiatric symptom severity, as assessed by the total Positive and Negative Syndrome Scale (PANSS) score. Secondary outcomes included social functioning measured by the Social Disability Screening Schedule (SDSS), behavioral and psychiatric symptoms evaluated by the Nurses’ Observation Scale for Inpatient Evaluation (NOSIE), and activities of daily living assessed by the Activities of Daily Living (ADL) scale. (5) Study type: Only publicly available RCTs were eligible for inclusion.

The exclusion criteria were as follows: (1) patients with severe hepatic and renal insufficiency and (2) studies with incomplete data or outcome measures from which data could not be extracted.

### 2.4 Study selection and data extraction

Study selection was independently performed by two reviewers (Sun and Teng), who screened the literature based on inclusion and exclusion criteria using NoteExpress software. Duplicated and irrelevant articles were first excluded based on their titles and abstracts. Thereafter, the full texts of potentially eligible articles were downloaded and reviewed to identify all eligible studies. Data extraction was independently performed by the two abovementioned reviewers (Sun and Zhang). Any disagreements were resolved by consulting the third reviewer (Song). The following data were extracted from each selected study: author, year, age, country, measures for the control group and the intervention group, diagnostic criteria, sample size, and outcomes.

### 2.5 Quality assessment

The Cochrane risk of bias tool was used to evaluate the risk of bias in the included RCTs (25). The tool assessed the following seven domains: random sequence generation, allocation concealment, blinding of participants and personnel, blinding of outcome assessment, incomplete outcome data, selective reporting, and other bias. Quality evaluation was independently performed by the two reviewers (Sun and Xu). If there was any disagreement, the third reviewer (Jiang) was consulted to reach a consensus.

### 2.6 Statistical analysis

Continuous variables were expressed as mean ± standard deviation (SD) and 95% confidence intervals (CIs). Statistical heterogeneity was assessed using the *I*^2^ statistic and the Cochrane *Q* test, with *I*^2^ indicating the percentage of total variation across studies. A fixed-effect model was used when *I*^2^ was ≤ 50% and *p* > 0.1, whereas a random-effects model was applied when *I*^2^ > 50% or *p* ≤ 0.1. Given the potential clinical heterogeneity inherent in occupational therapy interventions, the random-effects model was uniformly applied to all meta-analyses to provide a more conservative estimate. All statistical analyses were performed using Review Manager (RevMan) version 5.4 and R software (version 4.4.3).

## 3 Results

### 3.1 Study selection

**Figure 1:**
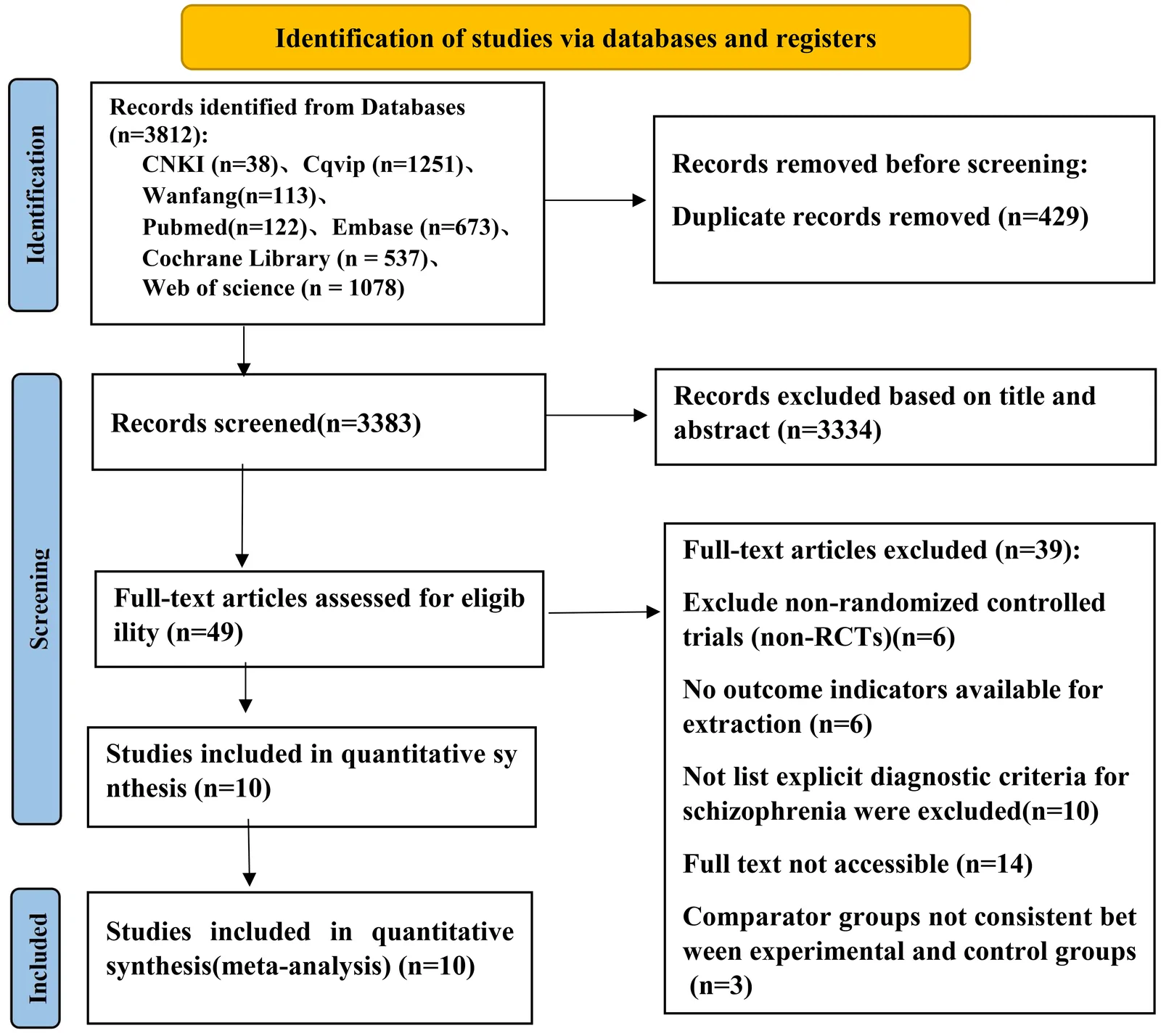
PRISMA flow diagram.

A total of 3,812 records were identified through electronic database searches. Following the removal of duplicates, 3,383 articles were screened by title and abstract. After title and abstract screening, 3,334 unrelated articles were excluded. Subsequently, 49 articles were assessed for full-text eligibility. Of these, 39 articles were excluded, including 10 because of unclear diagnostic criteria and six because they were not randomized controlled trials. Ultimately, 10 trials (26–35) were included (**Figure 1**).

### 3.2 Study characteristics

These trials were conducted between 2015 and 2025 in China and Japan. The sample sizes of the individual trials ranged from 40 to 200 participants. A total of 958 patients were included across all studies. The characteristics of the included trials are presented in **Supplementary Table 1**.

### 3.3 Study quality

**Figure 2:**
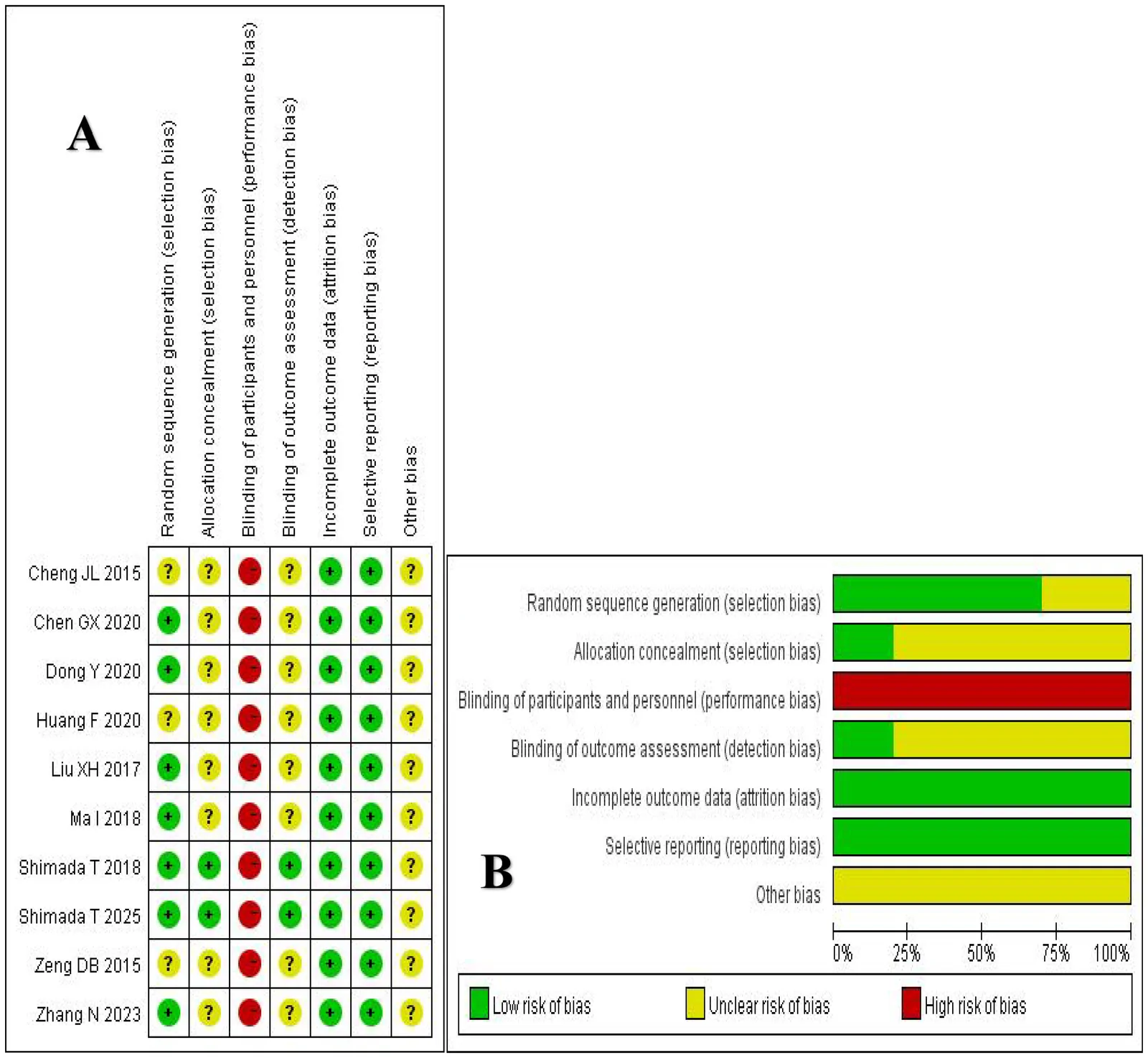
Risk of bias assessment for included studies. **(A)** Risk of bias summary plot; **(B)** Risk of bias graph.

All 10 RCTs reported the baseline characteristics of the participants. Regarding the randomization method (e.g., random number tables), seven studies were rated as having a “low risk” of bias because of adequate sequence generation, whereas the remaining three were rated as having an “unclear” risk for this domain. Blinding of participants and personnel was not feasible in occupational therapy interventions; therefore, the risk of performance bias was judged to be “high”. For detection bias, most studies were judged to be at “unclear” risk because of a lack of explicit reporting on the blinding of outcome assessors. A summary of the risk-of-bias assessment for each included trial is presented in **Figure 2**.

### 3.4 Meta-analysis

#### 3.4.1 Effects of occupational therapy interventions on PANSS total scores

**Figure 3:**
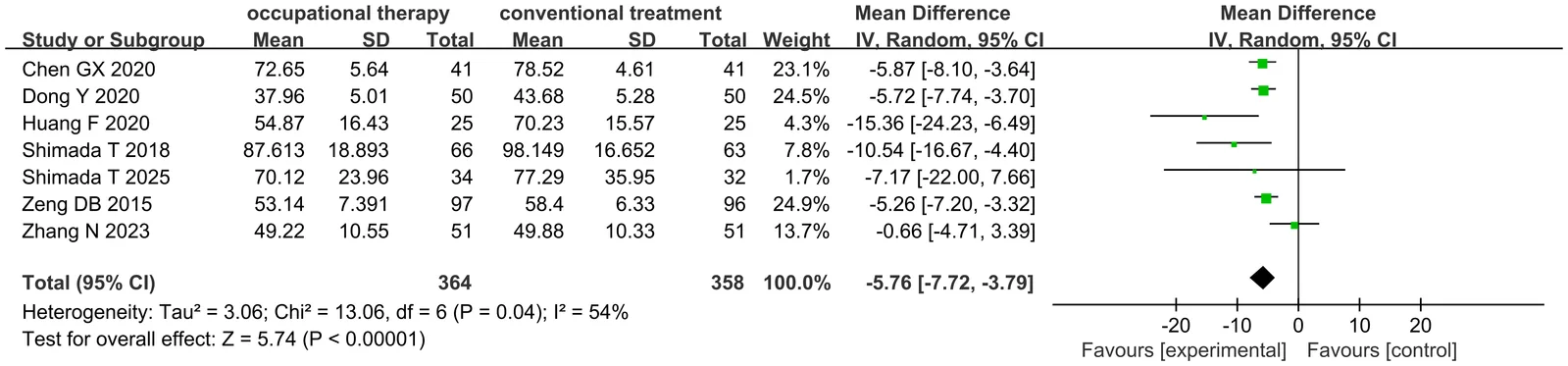
Forest plot for PANSS total scores.

The meta-analysis of seven trials (26, 27, 31–35) (*n* = 722), using the total PANSS score as the outcome measure, identified moderate heterogeneity (*I*^2^ = 54%; *p* = 0.04). Owing to this heterogeneity, a random-effects model was employed. The pooled analysis showed that the occupational therapy group had a significantly lower total PANSS score than the conventional treatment group (MD: − 5.76; 95% CI, − 7.72 to − 3.79; *p* < 0.00001; **Figure 3**).

#### 3.4.2 Effects of occupational therapy interventions on social function

**Figure 4:**
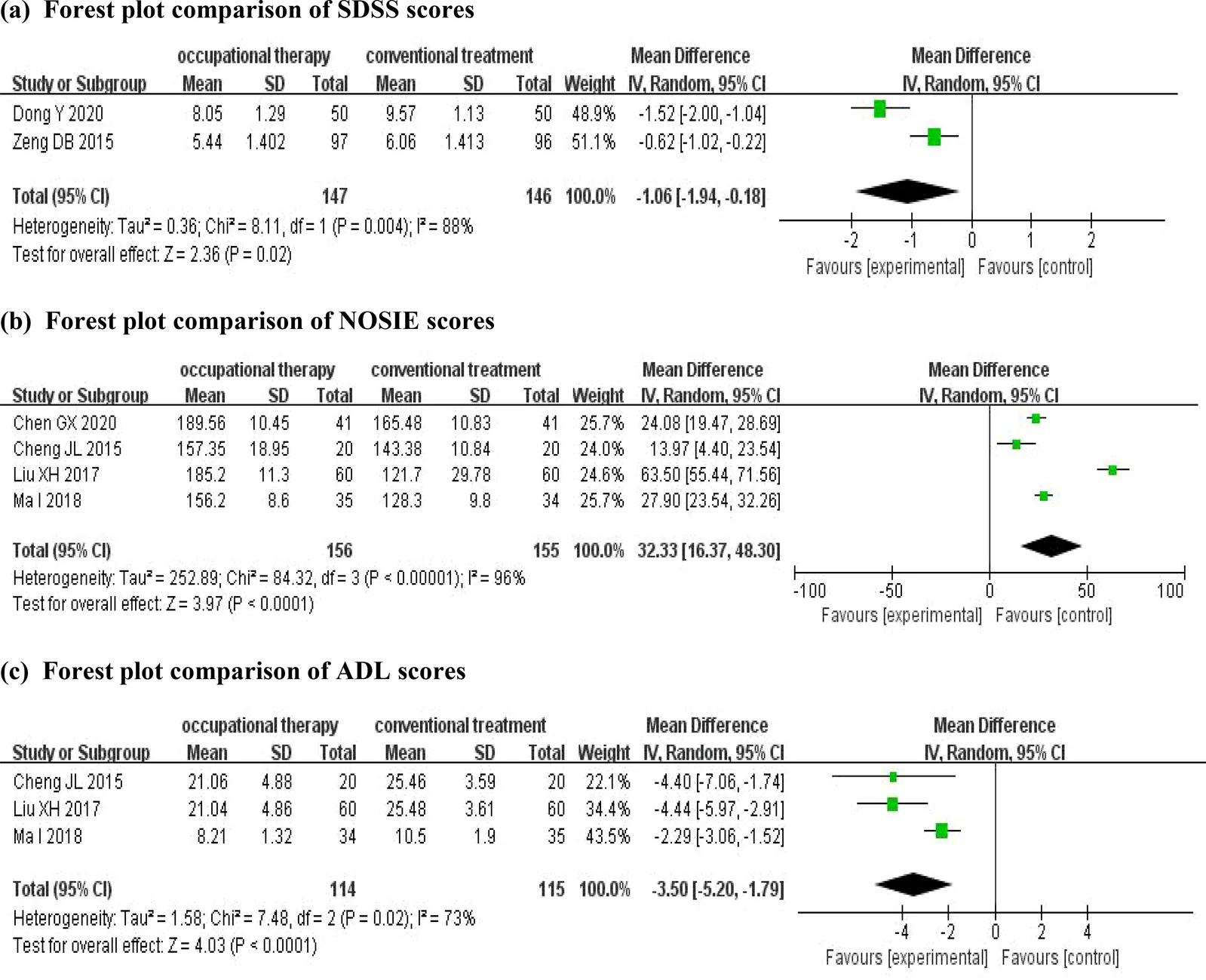
Forest plot summary of secondary outcomes. **(A)** Forest plot comparison of SDSS scores. **(B)** Forest plot comparison of NOSIE scores. **(C)** Forest plot comparison of ADL scores.

The meta-analysis of two trials (27, 33) (*n* = 293), using the SDSS score as the outcome measure, identified considerable heterogeneity (*I*^2^ = 88%; *p* = 0.004), prompting the use of a random-effects model. The pooled results showed that the occupational therapy group had a significantly lower SDSS score than the control group (MD: − 1.06; 95% CI, − 1.94 to − 0.18; *p* = 0.02; **Figure 4a**).

For the NOSIE, a meta-analysis of four trials (28–31) (*n* = 311) also revealed high heterogeneity (*I*^2^ = 96%; *p* < 0.00001), and a random-effects model was therefore applied. The pooled analysis showed that the occupational therapy group had a significantly higher NOSIE than the usual care group (MD: 32.33; 95% CI, 16.37 to 48.30; *p* < 0.0001; **Figure 4b**), suggesting that occupational therapy may improve the overall psychiatric behavioral status of patients with schizophrenia.

A meta-analysis of three trials (28–30) (*n* = 229), using the ADL score as the outcome measure, revealed substantial heterogeneity (*I*^2^ = 73%; *p* = 0.02), prompting the use of a random-effects model. The pooled analysis showed that the occupational therapy group had a significantly lower ADL score than the control group (MD: − 3.50; 95% CI, − 5.20 to − 1.79; *p* = 0.0001; **Figure 4c**), suggesting that occupational therapy may improve activities of daily living in patients with schizophrenia.

#### 3.4.3 Subgroup analysis of the PANSS total score

**Figure 5:**
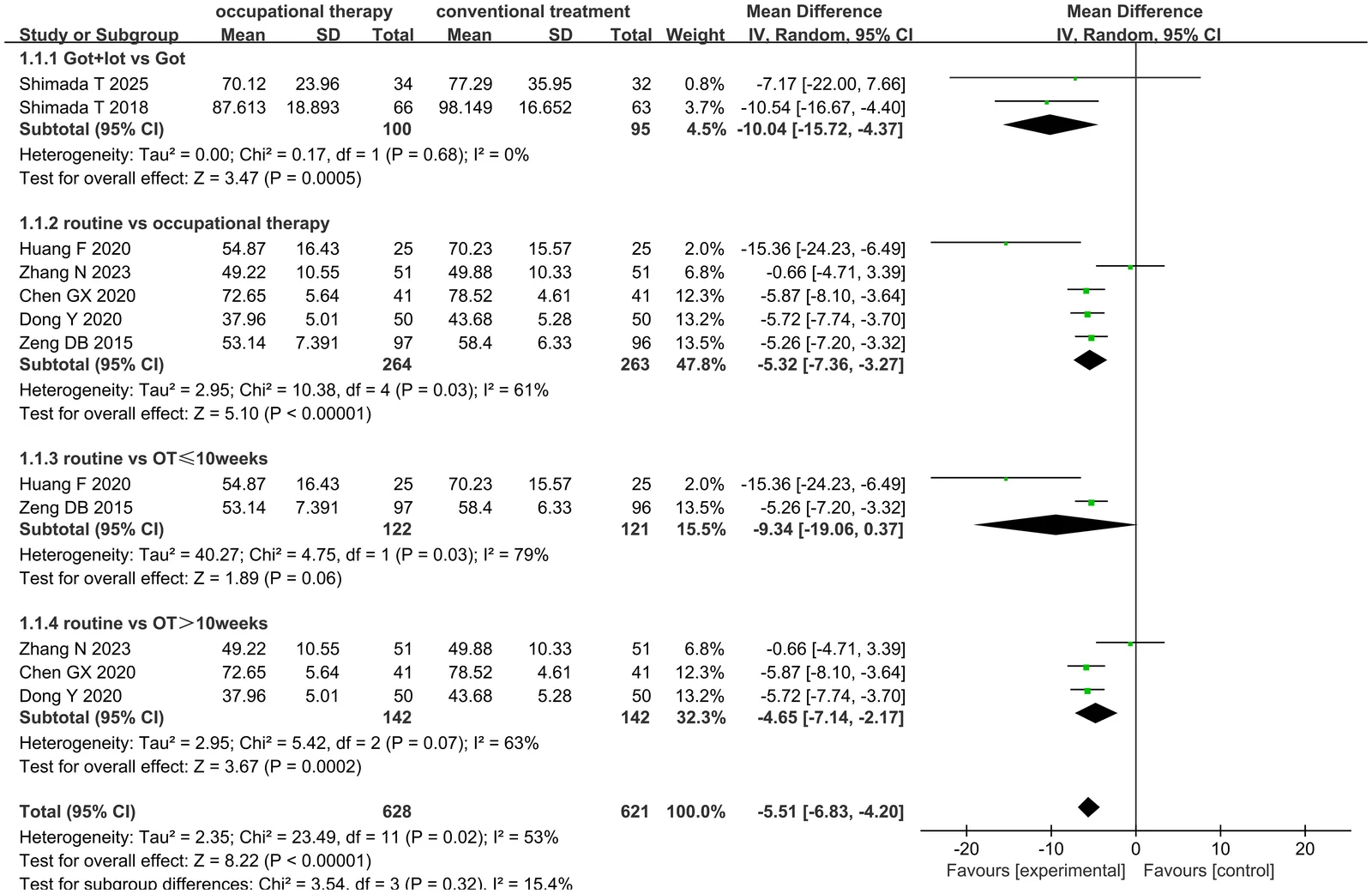
Forest plot of subgroup analyses for PANSS total score.

In the subgroup analysis stratified by intervention type, the Got + lot versus Got subgroup included two studies (*I*^2^ = 0%, MD: − 10.04; 95% CI, − 15.72 to 4.37; *p* = 0.0005), whereas the conventional treatment versus occupational therapy subgroup comprised five studies (*I*^2^ = 61%, MD: − 5.32; 95% CI, − 7.36 to − 3.27; *p* < 0.00001). Given that moderate heterogeneity was observed in the latter subgroup, a secondary subgroup analysis based on intervention duration was conducted. The ≤ 10-week subgroup (two studies) yielded an MD of − 9.34 (95% CI, − 19.06 to 0.37; *p* = 0.06) with an *I*^2^ of 79%, whereas the > 10-week subgroup (three studies) yielded an MD of − 4.65 (95% CI, − 7.14 to − 2.17; *p* = 0.0002) with an *I*^2^ of 63%. The test for subgroup differences yielded *χ*^2^ = 3.54 (*df* = 3; *p* = 0.32) (**Figure 5**), indicating that the pooled effects across the intervention duration subgroups did not differ significantly and should therefore be interpreted only as a numerical trend. Further group analyses based on large-sample, well-designed randomized controlled trials with standardized intervention durations are warranted to determine the potential impact of intervention length on the rehabilitative efficacy of occupational therapy.

#### 3.4.4 Sensitivity analysis and funnel plot analysis

**Figure 6:**
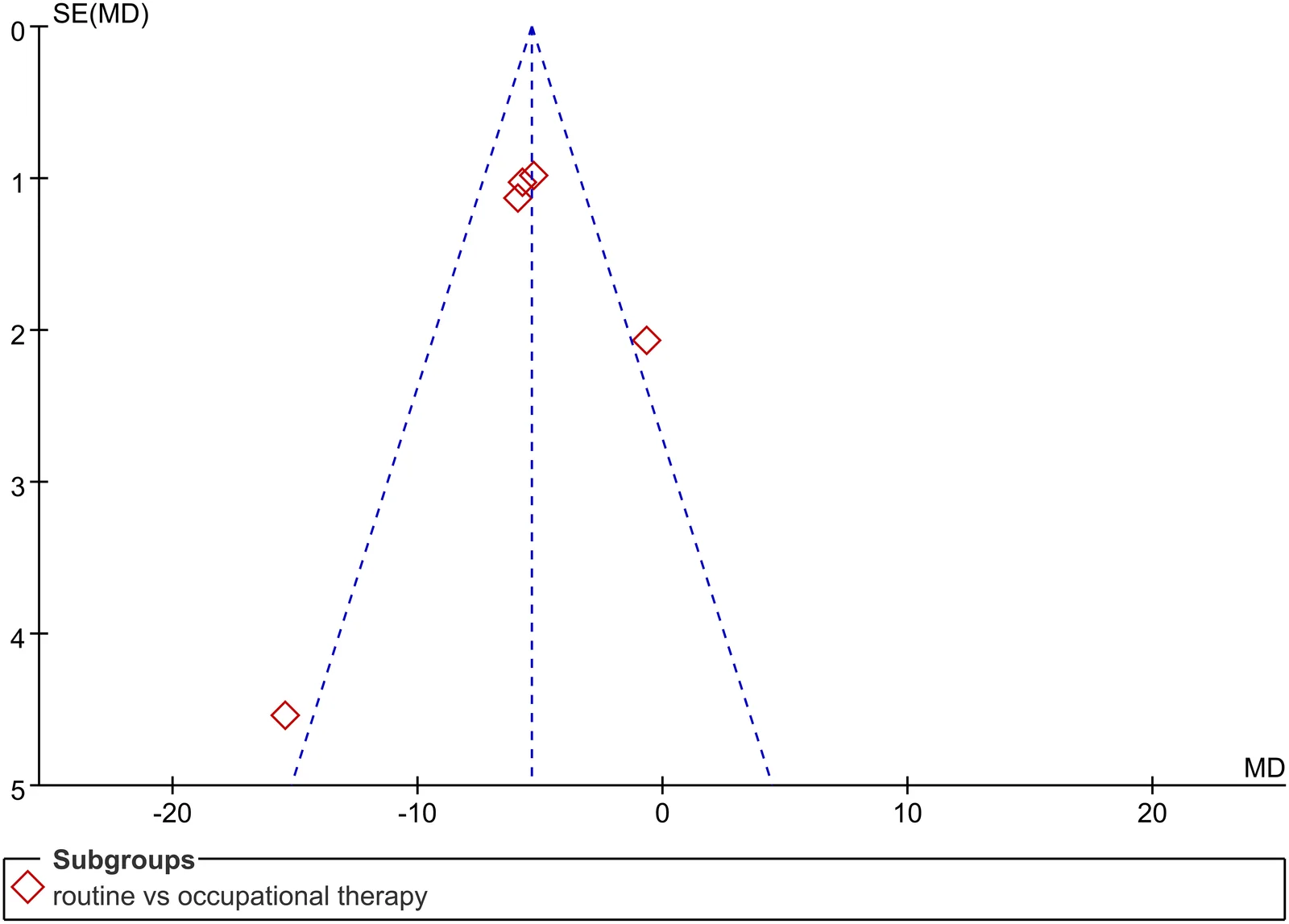
Funnel plot of publication bias for PANSS total score outcomes.

Sensitivity analysis was performed using the leave-one-out approach. The original pooled model yielded a Tau^2^ of 0.0009 and an *I*^2^ of 61.5%, indicating moderate between-study heterogeneity. Excluding Huang (26) or Zhang (32) individually resulted in no appreciable change in between-study variance. However, the exclusion of any one of Chen (31), Dong (33), or Zeng (27) increased Tau² to 12.68–13.55, with *I*^2^ exceeding 70%. In all single-study exclusion scenarios, the 95% confidence intervals of the pooled mean difference did not cross the null value, and all *p*-values were < 0.01. The pooled MD ranged from − 5.20 to − 5.76, which was highly consistent with that of the original random-effects model (MD: − 5.37; 95% CI, − 6.49 to − 4.24; *p* < 0.0001). The results are summarized in **Table 1**. The funnel plot suggested the possibility of publication bias (**Figure 6**).

**Table 1:** Sensitivity analysis for PANSS total score.

Omitted study	Mean difference (MD)	95% confidence interval (95% CI)	p-value	Between-study heterogeneity (Tau2)	Tau
Omitting Huang (2020)	− 5.2	[− 6.34; − 4.07]	< 0.0001	< 0.0001	0.0028
Omitting Zhang (2023)	− 5.76	[− 6.94; − 4.59]	< 0.0001	< 0.0001	0.0016
Omitting Chen (2020)	− 5.54	[− 9.58; − 1.50]	0.0072	12.677	3.5605
Omitting Dong (2020)	− 5.61	[− 9.72; − 1.49]	0.0075	13.1442	3.6255
Omitting Zeng (2015)	− 5.76	[− 9.93; − 1.60]	0.0067	13.5473	3.6807
Random effects model	− 5.37	[− 6.49; −4.24]	< 0.0001	< 0.0001	0.0009

## 4 Discussion

Schizophrenia is a chronic and complex psychiatric disorder with multifactorial etiology, encompassing genetic, neurodevelopmental, and psychosocial contributors. Its clinical presentation is characterized by positive and negative symptoms, cognitive impairment, and progressive social dysfunction, all of which significantly undermine quality of life and mental well-being while imposing a substantial caregiving burden on families and healthcare systems. Prolonged hospitalization often compounds these challenges, leading to social withdrawal and difficulties in community reintegration (36). Although conventional pharmacotherapy and recreational activities can partially alleviate psychotic symptoms, their effects are largely symptom-focused and do not adequately address the broader functional deficits essential for independent living. In contrast, occupational therapy places greater emphasis on functional restoration and social re-engagement, offering a more holistic rehabilitative approach (37, 38).

Accumulating evidence suggests that occupational therapy confers meaningful benefits in schizophrenia rehabilitation. As a nonpharmacological intervention, it targets key areas of functional impairment—including social disengagement, amotivation, and cognitive deficits—through structured task-oriented training, facilitated social interaction, and purposeful environmental engagement. By fostering a sense of accomplishment through task completion, occupational therapy may also alleviate negative symptoms, such as affective blunting and diminished volition, while reducing the behavioral impact of residual psychotic features (21, 39, 40). While several studies have reported positive effects of occupational therapy on both clinical symptoms and social functioning, marked heterogeneity in intervention protocols, outcome measures, and follow-up durations across trials has precluded a systematic synthesis of the overall efficacy. To address this gap, the present study systematically reviewed available RCTs in which occupational therapy served as the primary intervention for patients with schizophrenia. We conducted a comprehensive evaluation of psychiatric symptoms (assessed using the PANSS total score) and social functioning (measured using the SDSS, NOSIE, and ADL scales) to provide an updated evidence base for this intervention.

The forest plot summary revealed that, despite considerable heterogeneity in the implementation protocols of occupational therapy across the included studies, an overall trend toward improvement in various clinical outcomes was observed in patients with schizophrenia. The pooled analysis of psychiatric symptoms, based solely on the total PANSS score, showed that the occupational therapy group had significantly lower total PANSS scores than the control group, suggesting a beneficial effect on overall psychotic symptomatology. However, because none of the included studies reported the positive and negative subscale scores separately, we were unable to distinguish the differential effects of the intervention on these two symptom dimensions and therefore relied on the total score to assess overall efficacy. Thus, the present evidence does not support the conclusion that occupational therapy improves positive and negative symptoms separately. For the secondary outcomes related to social functioning, the results suggested that occupational therapy may improve patients’ activities of daily living. However, the credibility of this evidence is limited because the number of studies reporting SDSS, NOSIE, and ADL outcomes was small, and considerable heterogeneity was observed across these measures, likely reflecting variability in intervention standardization, baseline illness severity, and assessment time points.

In the subgroup analysis based on intervention type, the subgroup comparing occupational therapy plus conventional treatment with conventional treatment alone included only a limited number of studies and failed to detect a statistically significant difference, which may be attributable to the small sample size and limited statistical power. The subgroup comparing occupational therapy alone with conventional treatment showed moderate heterogeneity, prompting further stratification according to intervention duration. The short-term intervention subgroup (≤ 10 weeks) did not reach statistical significance, whereas the longer-term subgroup (> 10 weeks) demonstrated significant rehabilitative benefits. However, the test for subgroup differences was not statistically significant, indicating that the pooled effects between short- and long-term interventions did not differ significantly. Therefore, these results should be interpreted as a numerical trend rather than evidence of a true subgroup effect. We cannot conclude that short-term interventions lack rehabilitative benefit or that longer intervention durations confer superior efficacy. Large-sample, well-designed randomized controlled trials with standardized intervention durations are warranted to determine whether intervention length moderates therapeutic efficacy.

The leave-one-out sensitivity analysis indicated that the overall pooled model had a moderate level of heterogeneity and that fluctuations in heterogeneity were directly associated with the degree of standardization in intervention implementation details across the included studies. Excluding the two studies with comparable protocols (Huang and Zhang) did not appreciably alter the between-study variance. However, removing any of the three studies (Chen, Dong, and Zeng) increased both between-study variance and the overall heterogeneity. This finding may be explained by the baseline clinical data presented in **Supplementary Table 1**: although the included studies evaluated similar occupational therapy domains, there were differences in intervention providers, treatment frequency, session duration, and total rehabilitation course. The three standardized studies were all delivered by uniformly trained healthcare personnel with complete and standardized documentation of training cycles and intensity, which may have helped offset the variability in intervention implementation across the remaining studies. In contrast, Huang was conducted exclusively by a full-time occupational therapist, representing a distinct execution subgroup, whereas Zhang lacked key information regarding treatment course and frequency. Consequently, neither study effectively balanced the between-study variability, and excluding either study did not substantially affect the overall heterogeneity. These findings also suggest that the present meta-analysis was somewhat dependent on the three standardized procedures, indicating limited local stability. Although heterogeneity fluctuated notably following the exclusion of individual studies, the direction of the intervention effect did not reverse under any single-study exclusion scenario, and the pooled effect estimates remained highly consistent with those of the original random-effects model. These findings suggest that the conclusion that occupational therapy may improve psychiatric symptoms is robust.

Compared with previous systematic reviews, the present study broadened the range of outcome measures and incorporated subgroup analyses according to intervention type and duration, thereby expanding the existing evidence base. Collectively, the pooled data suggest that occupational therapy may serve as an adjunctive rehabilitative intervention for schizophrenia, with potentially beneficial effects on overall psychiatric symptoms and social functioning.

This study has several limitations that may affect the interpretation of the findings. First, the meta-analysis included only 10 randomized controlled trials, with some subgroups containing only two to three studies, and the limited sample size made it difficult to completely rule out the influence of publication bias. Second, regarding the risk of bias, because occupational therapy is a practical intervention, blinding of participants and therapists was not feasible, resulting in a high risk of performance bias. Moreover, most studies provided insufficient information on random sequence generation and allocation concealment, whereas only the outcome assessment and incomplete data domains were judged to be at relatively low risk of bias. Furthermore, considerable heterogeneity was observed across outcome measures, particularly for the NOSIE and SDSS indices, which may be attributable to the small number of included studies, as well as variations in patient characteristics, intervention protocols, and study designs. Third, the included studies were predominantly single-center, small-sample trials conducted mainly in Asia, with a primary focus on short-term efficacy and limited long-term follow-up data (≥ 6 months or 1 year or more), making it difficult to evaluate the impact of occupational therapy on long-term prognosis. Collectively, the strength of evidence from this study is limited, and the findings should be interpreted in the context of the clinical circumstances. Future large-scale, multicenter randomized controlled trials with standardized interventions, long-term follow-up, and rigorous methodology are warranted to further validate the rehabilitative effects of occupational therapy in patients with schizophrenia.

## 5 Conclusion

Occupational therapy-based interventions may contribute to improvements in psychiatric symptoms and appear to exert positive effects on social functioning and activities of daily living in patients with schizophrenia. Although the current evidence has certain limitations, the overall findings tentatively suggest that occupational therapy has potential as a rehabilitative intervention. However, further refinement of standardized protocols is needed to facilitate its structured implementation in mental health settings.
