# Supplementary material for: Effects of occupational therapy on psychiatric symptoms and social functioning in patients with schizophrenia: a systematic review and meta-analysis
Source: Front Psychiatry. 2026 Aug 12;17:1862106. doi: 10.3389/fpsyt.2026.1862106 (PMC13508030; doi:10.3389/fpsyt.2026.1862106)
Supplement: Supplementary file 1 [file DataSheet1.pdf]

Supplementary Table 1 Characteristics of the included trials

Source	Country	Control group measures	Intervention group measures	Diagnostic criteria	Intervention providers	Intervention frequency	Duration (weeks)	Group size/age		Outcomes
								CG	IG	
Huang F 2020	China	CT	ADL、IADL、education、work、play、leisure、social participation.	CCMD-3	occupational therapists	5times/weeks、30min/times	10	25/42.60±6.70	25/42.90±5.40	①
Zeng DB 2015	China	CT	ADL、IADL、rest and sleep、education、work、play、leisure、social participation.	DSM-4	trained healthcare professionals	5times/weeks、2h/times	8	100/ N/S	100/ N/S	①②
Chen GX 2020	China	CT	ADL、work、social participation.	CCMD-3	trained healthcare professionals	N/S	N/S	41/32.36±5.16	41/33.56±3.86	①③
Zhang N2023	China	CT	ADL、IADL、education、work、play、leisure、social participation.	ICD-10	trained healthcare professionals	N/S	12	51/37.88±5.30	51/38.80±5.70	①

DongY 2020	China	CT	ADL、IADL、rest and sleep、education、work、play、leisure、social participation.	ICD-10	trained healthcare professionals	3times/weeks、45min/times	20	50/39.14±5.41	50/38.54±5.25	①②
Shimada T 2018	Japan	GOT	IOT+GOT	DSM-5	occupational therapists	3-5times/weeks、1-2h/times	12	66/43.39 ±9.98	63/41.39±11.04	①
Shimada T 2025	Japan	GOT	IOT+GOT	DSM-5	occupational therapists	3-5times/weeks、1-2h/times	12	34/43.47 ±9.59	32/44.97 ±9.17	①
Ma L 2018	China	CT	education、work、play、leisure、social participation.	CCMD-3	trained nursing staff	N/S	24	35/40.50±3.30	34/40.30±2.50	③④
Cheng JL2015	China	CT	IADL、education、work、play、leisure、social participation.	CCMD-3	trained healthcare professionals	N/S	48	20/42. 90 ± 5. 40	20/42. 60 ± 6. 70	③④
Liu XH 2017	China	CT	ADL 、 IADL 、education、work、play、leisure、social participation.	CCMD-3	occupational therapists	2times/days、45min/times	N/S	60/42.40± 6.90	60/42.70 ± 5.30	③④

Note：①Positive and Negative Syndrome Scale(PANSS)②Social Disability Screening Schedule(SDSS)③Nurses' Observation Scale for Inpatient Evaluation(NOSIE)④Activities of Daily Living (ADL) IG, intervention group; CG, control group; CT, Conventional treatment; IOT, motivational interviews, self-monitoring, individualized visits, craft activities, individualized psychoeducation, and discharge planning; GOT, physical fitness, craft activities, cooking, music, recreation, and psychoeducation. Intervention activities of GOT and IOT fall within the eight AOTA occupational domains.

The specific intervention content for each domain is as follows: (1) Activities of daily living (ADL) – training in eating, dressing, bathing, grooming, bed-making, organizing personal belongings, nail trimming, and the cultivation of personal hygiene habits; (2) Instrumental activities of daily living (IADL) – household management (e.g., floor mopping, laundry, and general cleaning), training in home cooking and dessert preparation, standardized medication management, allocation of harvested crops and material resources, as well as planning for the exchange and sale of labor products; (3) Health management – comprehensive occupational therapy program planning and long-term post-discharge rehabilitation follow-up, psychoeducation and interventions to enhance treatment adherence, motivational interviewing and self-monitoring behavioral training, emotional counseling and alleviation of anxiety and depression, targeted cognitive training and management of impulsive behaviors, positive reinforcement of adaptive behaviors and correction of maladaptive habits, and individual psychological support alongside group-based emotional counseling; (4) Rest and sleep – circadian rhythm regulation, optimization of the sleep environment, and sleep behavior modification; (5) Education – systematic instruction on the principles, objectives, procedural standards, and precautions of occupational therapy, specialized training in agricultural planting, health education lectures, traditional culture courses, and teaching of handicrafts and cooking skills; (6) Work – weeding, vegetable planting, watering, field management, crop harvesting, craft production (e.g., hand-weaving, ceramic clay modeling, and embroidery), on-site distribution and sales to hospital staff and the general public, and coordinated allocation of labor earnings and value transformation of vocational outputs; (7) Leisure – calligraphy, painting, hand-embroidery, board games, mahjong, card games, singing, dancing, music appreciation, movie viewing, ball sports, group outings, and team-based game competitions; (8) Social participation – group training, ice-breaking activities, scenario-based social simulation performances, peer-to-peer communication training, collective farming, off-site excursions to expand social contexts, and group psychological mutual-support sessions.
