# Supplementary material for: Effects of occupational therapy on psychiatric symptoms and social functioning in patients with schizophrenia: a systematic review and meta-analysis
Source: Front Psychiatry. 2026 Aug 12;17:1862106. doi: 10.3389/fpsyt.2026.1862106 (PMC13508030; doi:10.3389/fpsyt.2026.1862106)
Supplement: Supplementary file 2 [file DataSheet2.pdf]

The detailed search strategies for each database are presented as follows, with the search period limited to January 2025 to February 2026.

万方：(题名:精神分裂症 OR 题名:精神分裂症样障碍 OR 关键词:精神分裂症 OR 关键词:精神分裂症样障碍) AND (题名:作业疗法 OR 题名:作业治疗 OR 题名:职业疗法 OR 题名:职业治疗 OR 关键词:作业疗法 OR 关键词:作业治疗 OR 关键词:职业疗法 OR 关键词:职业治疗)

维普：(((((((题名或关键词=精神分裂症 OR 题名或关键词=post schizophrenia depression) OR 题名或关键词=schizophrenia) OR 题名或关键词=schizophrenic) OR 题名或关键词=schizophrenic patients) OR 题名或关键词=schizophrenics) OR 题名或关键词=早发性痴呆) AND ((((题名或关键词=作业疗法 OR 题名或关键词=occupational therapy) OR 题名或关键词=work therapy) OR 题名或关键词=作业治疗) OR 题名或关键词=职能治疗)) AND (years:[2015 TO 2026])

知网：（主题：精神分裂症）AND（主题：作业疗法）

PubMed : (schizophrenia[MeSH Terms] OR schizophrenia[Title/Abstract] OR schizophreniform disorder[Title/Abstract]) AND (Occupational Therapy[MeSH Terms] OR occupational therapy[Title/Abstract] OR work therapy[Title/Abstract]) AND 2015:2026[dp].

Web of science ： TS=(schizophrenia OR schizophreniform disorder) AND TS=(occupational therapy OR work therapy OR occupational rehabilitation)

Cochrane library ： #1 Schizophrenia #2 Schizophreniform Disorder #3 schizophrenia* #4 schizophrenifor* #5 #1 OR #2 OR #3 OR #4 #6 Occupational Therapy #7 occupational therap* #8 work therap* #9 #6 OR #7 OR #8 #10 #5 AND #9

Embase ： ('schizophrenia'/exp OR 'schizophrenia' OR 'schizophreniform disorder') AND ('occupational therapy'/exp OR 'occupational therapy' OR 'work therapy') AND [2015-2025]/py
